# Влияние дистанционного мониторинга самоконтроля гликемии на углеводный обмен и качество жизни пациентов с сахарным диабетом 1 типа

**DOI:** 10.14341/probl13535

**Published:** 2024-11-02

**Authors:** Л. А. Суплотова, О. О. Алиева, Л. И. Ибрагимова

**Affiliations:** Тюменский государственный медицинский университет; Тюменский государственный медицинский университет; Национальный медицинский исследовательский центр эндокринологии

**Keywords:** сахарный диабет 1 типа, самоконтроль гликемии, дистанционный мониторинг, глюкометр, качество жизни

## Abstract

**ОБОСНОВАНИЕ:**

ОБОСНОВАНИЕ. Самоконтроль гликемии (СКГ) — основной инструмент в достижении целевых показателей углеводного обмена. Дистанционный мониторинг СКГ в России появился относительно недавно и нуждается в оценке эффективности.

**ЦЕЛЬ:**

ЦЕЛЬ. Оценить влияние дистанционного мониторинга СКГ на углеводный обмен и качество жизни пациентов с сахарным диабетом  1 типа (СД1) с целью формирования новых терапевтических подходов.

**МАТЕРИАЛЫ И МЕТОДЫ:**

МАТЕРИАЛЫ И МЕТОДЫ. Пациенты с СД1 с гликированным гемоглобином (HbA1c) от 8,0 до 12,0% были разделены на основную (n=107) и контрольную группы (n=20). Пациенты из основной группы проводили СКГ при помощи глюкометров с возможностью дистанционной передачи данных, пациенты из группы контроля продолжали традиционный СКГ. Оценивалась динамика HbA1c, расчетные показатели времени нахождения в целевых диапазонах, распознавание гипогликемии (шкала GOLD, опросник Clarke), качество жизни по опроснику SF-36. Статистический анализ проводился в программе SPSS Version 26.0 (IBM, США).

**РЕЗУЛЬТАТЫ:**

РЕЗУЛЬТАТЫ. В основной группе (n=88) отмечалось статистически значимое снижение HbA1c через 6 месяцев с 9,0% [8,4; 9,9] до 8,1% [7,4; 9,2] (p<0,001), при СКГ более 4 раз в сутки — до 7,3% [7,0; 7,8] (p=0,001). В группе контроля (n=20) к 6-му месяцу HbA1c вырос до 10,1% [8,9; 11,2] (p=0,010). Продемонстрировано достоверное увеличение в основной группе расчетного времени в целевом диапазоне, derived Time In Range, до 69,9±13,0 (95% ДИ 65,73–74,03; p<0,001); расчетное время в диапазоне выше целевого, derived Time Above Range, достоверно снизилось до 9,5% [6,4; 15,0] (p<0,001), расчетное время в диапазоне ниже целевого, derived Time Below Range, — до 6,7% [2,8; 12,2] (p=0,044); коэффициент вариабельности, Coefficient of Variation, достиг 36,3±7,9 (95% ДИ 33,7–38,8; p<0,001). По результатам SF-36, в основной группе значимо улучшились физический и психологический компоненты качества жизни (p<0,001). Распознавание гипогликемии улучшилось в группе вмешательства (-4,5% пациентов (p=0,046) по опроснику Clarke; -8% (p=0,008) по шкале GOLD).

**ЗАКЛЮЧЕНИЕ:**

ЗАКЛЮЧЕНИЕ. Дистанционный мониторинг СКГ является перспективным терапевтическим подходом ввиду положительного влияния на углеводный обмен и качество жизни пациентов с СД1.

## Abstract (en)

**BACKGROUND:**

BACKGROUND: Self-monitoring of blood glucose (SMBG) is the main tool to achieve carbohydrate metabolism targets in patients with type 1 diabetes mellitus (DM). Remote monitoring of SMBG in Russia appeared relatively recently and needs to be evaluated for effectiveness.

**AIM:**

AIM: To evaluate the effect of remote monitoring of SMBG on carbohydrate metabolism and quality of life in patients with type 1 DM in order to form new therapeutic approaches.

**MATERIALS AND METHODS:**

MATERIALS AND METHODS: Patients with type 1 DM with glycated hemoglobin (HbA1c) from 8.0 to 12.0% were divided into the main (n=107) and control group (n=20). Patients from the main group performed SMBG using glucometers with the possibility of remote data transmission, patients from the control group continued the traditional SMBG. The dynamics of HbA1c, derived time spent in the target ranges, recognition of hypoglycemia (GOLD scale, Clarke questionnaire), quality of life according to the SF-36 questionnaire were evaluated. The statistical analysis was carried out in the SPSS Version 26.0 program (IBM, USA).

**RESULTS:**

RESULTS: In the main group (n=88) the HbA1c was statistically significant decreased after 6 months from 9.0% [8.4; 9.9] to 8.1% [7.4; 9.2] (p<0.001), with SMBG more than 4 times a day - up to 7.3% [7.0; 7.8] (p=0.001). In the control group (n=20), by the 6th month, HbA1c increased to 10.1% [8,9; 11,2] (p=0,010). Derived Time In Range in the main group increased to 69.9±13.0 (95% CI 65.73–74.03; p<0.001); derived Time Above Range significantly decreased to 9.5% [6.4; 15.0] (p<0.001), derived Time Below Range — to 6.7% [2.8; 12.2] (p=0.044); Coefficient of Variation reached 36.3±7.9 (95% CI 33.7–38.8; p<0.001). According to the results of SF-36, the physical and psychological components of the quality of life in the main group significantly improved (p<0.001). Recognition of hypoglycemia improved in the intervention group (-4.5% of patients (p=0.046) according to the Clarke questionnaire; -8% (p=0.008) on the GOLD scale).

**CONCLUSION:**

CONCLUSION: Remote monitoring of SMBG is a prospective therapeutic approach due to its positive effect on carbohydrate metabolism and quality of life in patients with type 1 DM.

## ОБОСНОВАНИЕ

По данным Федерального регистра сахарного диабета (СД) по итогам 2022 г., несмотря на возможности современных методов контроля углеводного обмена, остается высокой доля пациентов с СД 1 типа (СД1), не достигающих индивидуальных целевых показателей гликированного гемоглобина (HbA1c). Доля пациентов с HbA1c выше 8,0% составляет 37,9%, из них 20,4% пациентов с HbA1c выше 9,0% [1]. HbA1c позволяет оценить эффективность терапии СД и напрямую ассоциирован с риском развития осложнений и долгосрочным прогнозом.

На сегодняшний день существует широкое разнообразие средств контроля гликемии, наиболее современными являются системы непрерывного мониторирования глюкозы (НМГ). Однако на настоящий момент только 30% пациентов с СД1 используют НМГ. В то время как НМГ имеет множество явных преимуществ для людей с СД1, их использование сопряжено с рядом проблем [2][3].

По результатам опроса пациентов с СД1, наиболее часто упоминаемой категорией сложностей в использовании НМГ была их стоимость (61%), далее следовали проблемы, связанные с неудобством и непринятием необходимости носить прибор на теле (58,6%) [4]. Распространенным источником ошибок при использовании НМГ является так называемый «артефакт сдавления», который часто возникает во время сна. В одном исследовании, проведенном в Сан-Франциско, было отмечено, что аберрантные показания НМГ более 25 мг/дл от медианы возникали в определенных позах во время сна, которые, как считалось, сжимают локальную ткань вокруг сенсора и уменьшают приток крови [5]. Ложно низкие значения глюкозы как правило сопровождаются оповещением от сенсора НМГ, которое предупреждает об опасности. Это может разбудить и напугать пациента, а также привести к ошибочному решению о необходимости приема быстроусвояемых углеводов. Также начало работы нового датчика сопровождается периодом разогрева, и показания НМГ могут быть менее точными в первые 24 часа [6]. Большинство систем НМГ основаны на специфической электрохимической методологии измерения глюкозы в интерстициальной жидкости. Ряд эндогенных веществ, лекарственных препаратов и компоненты пищи, циркулирующие в организме человека, также могут влиять на измерения, проводимые системами НМГ, то есть сдвигать данные о «глюкозе» в ту или иную сторону [7].

Самоконтролю гликемии (СКГ) с помощью глюкометра исторически принадлежит основополагающее значение в достижении целевых показателей HbA1c. СКГ имеет решающее значение для оптимизации безопасности и эффективности сложных схем инсулинотерапии. В связи с этим, согласно клиническим рекомендациям, пациентам необходимо продолжать СКГ с помощью глюкометра не реже 4 раз в сутки при использовании НМГ и не реже 2 раз в сутки при использовании флэш-мониторинга глюкозы [8]. Современные глюкометры соответствуют критериям точности ГОСТ Р ИСО 15197–2015: «95 % измеренных значений глюкозы должны находиться в пределах или ± 0,83 ммоль/л (±15 мг/дл) среднего измеренных значений референтной методикой выполнения измерения при концентрации глюкозы <5,55 ммоль/л (<100 мг/дл) или в пределах ±15% при концентрации глюкозы >5,55 ммоль/л (>100 мг/дл)» [9].

По прогнозам, в 2024 г. число пользователей смартфонов во всем мире составит 4,88 млрд, это означает, что 60,42% населения мира владеет смартфонами [10]. Новым витком в развитии СКГ стала разработка технологии дистанционного мониторинга данных с глюкометра с использованием смартфона пациента (такие системы, как iBGStar, The Glucoonline system, mySugr, Livongo). Первый российский глюкометр с функцией удаленной передачи данных «Сателлит Online» получил регистрационное удостоверение в декабре 2022 г. [11]. Также в Российской Федерации доступны такие системы дистанционного контроля гликемии с помощью глюкометра, как мобильное приложение CONTOUR DIABETES, совместно с глюкометром Contour Plus One и мобильное приложение OneTouch Reveal совместно с глюкометрами OneTouch Verio Reflect и OneTouch Select Plus Flex. Согласно данным метаанализа, использование мобильного приложения с калькуляторами инсулина и углеводов показали очевидные преимущества улучшения HbA1c у молодых пациентов с СД1. Поскольку смартфоны все чаще используются в повседневной жизни, мобильные приложения могут стать потенциальной стратегией для улучшения показателей углеводного обмена у пациентов с СД [12]. Преимуществом данного метода является его доступность широким слоям населения, в том числе в сельской местности (при наличии стабильного интернет-соединения), возможность удаленного доступа врача-эндокринолога к данным дневника СКГ в реальном времени и проведения своевременной консультации при необходимости.

Таким образом, дистанционный мониторинг СКГ является актуальным вопросом, требующим изучения. В частности, интерес представляет анализ влияния на углеводный обмена и качество жизни пациентов с СД по сравнению с традиционным СКГ.

## ЦЕЛЬ ИССЛЕДОВАНИЯ

Оценить влияние дистанционного мониторинга СКГ на углеводный обмен и качество жизни пациентов с СД1 с целью формирования новых терапевтических подходов.

## МАТЕРИАЛЫ И МЕТОДЫ

## Место и время проведения исследования

Набор участников исследования проводили с ноября 2022-го по декабрь 2023 гг. в шести поликлиниках г. Тюмени: ГАУЗ ТО «Городская поликлиника №5», ГАУЗ ТО «Городская поликлиника №8», ГБУЗ ТО «Областная клиническая больница №2», ГАУЗ ТО «Городская поликлиника №3», ГАУЗ ТО «Городская поликлиника №12», ГАУЗ ТО «Городская поликлиника №17».

## Изучаемые популяции

Участниками настоящего исследования были пациенты с СД1 на дистанционном мониторинге СКГ и на традиционном СКГ (группа контроля).

Критерии включения:

1) подписание добровольного информированного согласия;

2) СД1 на режиме многократных инъекций инсулина;

3 )возраст от 18 до 70 лет;

4) длительность заболевания более 1 года;

5) HbA1c от 8,0 до 12,0%;

6) наличие данных HbA1c давностью менее 1 месяца от старта исследования.

Критерии невключения:

1) другие типы СД;

2) возраст менее 18 и более 70 лет;

3) длительность заболевания менее 1 года;

4) помповая инсулинотерапия;

5) отсутствие технической возможности дистанционной передачи результатов СКГ.

Критерии исключения:

1) отзыв информированного добровольного согласия;

2) неустранимые технические проблемы, связанные с оборудованием для проведения дистанционного мониторинга СКГ или с его использованием пациентом.

## Дизайн исследования

Проведено многоцентровое интервенционное динамическое проспективное исследование, в котором в соответствии с общими критериями включения были сформированы основная и контрольная группы. В основную группу на дистанционном мониторинге СКГ вошли 107 человек, а в группу контроля на традиционном СКГ — 20 человек с СД1. Все участники исследования подписали информированное добровольное согласие и находились под наблюдением в течение 6 месяцев.

Протокол исследования включал в себя два этапа, на первом из которых всем пациентам проводили клиническое и лабораторное обследование. Пациентам основной группы (n=107) компанией «ЭЛТА» были предоставлены глюкометры «Сателлит Online» с поддержкой технологии дистанционной передачи данных, а пациентам группы контроля — глюкометры «Сателлит Экспресс». Участники обеих групп были в равной степени обеспечены тест-полосками в зависимости от рекомендуемой частоты СКГ в соответствии с клиническими рекомендациями [13].

Пациенты основной группы использовали мобильное приложение для синхронизации по Bluetooth данных СКГ и введения маркеров событий (прием пищи, физическая нагрузка, прием лекарственных препаратов, инъекции инсулина). Данные из мобильного приложения поступали в личный кабинет врача-эндокринолога на vdiabete.com. Врач, в свою очередь, еженедельно анализировал данные СКГ, принимал решение о необходимости очного приема, телеконсультации и коррекции терапии. Схема передачи данных СКГ представлена на рисунке 1. Пациенты контрольной группы продолжали традиционный СКГ, посещали врача-эндокринолога 1 раз в 3 месяца согласно клиническим рекомендациям [13].

**Figure:**
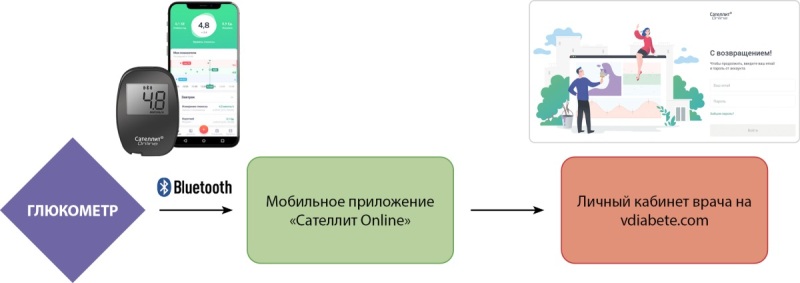
Рисунок 1. Схема передачи данных СКГ от пациента врачу-эндокринологу.

Для оценки качества жизни пациенты обеих групп заполняли валидированные опросники SF-36 Health Status Survey [14], для оценки распознавания гипогликемий (шкала GOLD [15], опросник Clarke [16]) на старте исследования и через 6 месяцев.

На втором этапе исследования проводилась оценка динамики качества контроля углеводного обмена: HbA1c; расчетного времени в целевом диапазоне, derived Time In Range (dTIR): 3,9–10 ммоль/л; расчетного времени в диапазоне выше целевого, derived Time Above Range (dTAR): более 10 ммоль/л; расчетного времени в диапазоне ниже целевого, derived Time Below Range (dTBR): менее 3,9 ммоль/л. Для анализа вариабельности гликемии рассчитывали коэффициент вариабельности, Coefficient of Variation (CV).

## Методы

Определение уровня HbA1с выполнялось в центральной клинико-диагностической лаборатории на базе ГБУЗ ТО «Областная клиническая больница №1» г. Тюмени, основывалось на методе определения (метод рефрактометрии на автоматическом анализаторе NicoCard Reader, Axis-Shield (Швеция)), сертифицированном в соответствии с Национальной программой стандартизации (National Glycohemoglobin Standardization Program — NGSP).

СКГ пациенты основной группы проводили при использовании индивидуального глюкометра «Сателлит Online», соответствующего ГОСТ Р ИСО 15197–2015 по аналитической и клинической точности. Пациентам было рекомендовано проведение СКГ 6 раз и более в сутки (натощак, постпрандиально, ситуационно). Еженедельно в рабочее время врач-эндокринолог и его медицинская сестра заходили в личный кабинет на vdiabete.com, просматривали аккаунты пациентов на наличие актуальных данных СКГ, оценивали наличие критических значений, время в целевых диапазонах гликемии, данные дневника самоконтроля. Врач принимал решение о необходимости проведения телеконсультации или очного приема, коррекции медикаментозной терапии. В случае достижения целевых показателей гликемии пациент посещал врача-эндокринолога 1 раз в 3 месяца. При отсутствии актуальных данных СКГ и невозможности решения проблемы по телефону или посредством телеконсультации пациента приглашали на очный прием врача или к техническому специалисту поликлиники (при наличии), а также передавали данные пациента в службу поддержки компании «ЭЛТА».

Для расчета показателей времени нахождения в диапазонах гликемии, на основании данных СГК (dTIR, dTAR, dTBR) использовалась программа Microsoft Excel 2016 с внесением следующих формул: dTAR=количество измерений глюкозы капиллярной крови более 10,0 ммоль/л/количество общих измерений глюкозы капиллярной крови *100%; dTBR=количество измерений глюкозы капиллярной крови менее 3,9 ммоль/л/количество общих измерений глюкозы капиллярной крови *100%; dTIR=100-%dTAR-%dTBR. Коэффициент вариации (CV) рассчитывался при использовании формулы: CV=SD/средняя гликемия*100%.

Заполняемый пациентами неспецифический опросник по качеству жизни «SF-36 Health Status Survey» состоит из 36 пунктов, которые сформированы в восемь шкал, они в свою очередь формируют два показателя: душевное и физическое благополучие (психологический и физический компонент здоровья). Результаты представляются в виде оценок в баллах (от 0 до 100), более высокая оценка соответствует более высокому уровню качества жизни [13]. Опросник Clarke — анкета из 8 вопросов о перенесенных эпизодах гипогликемии, пороге их распознавания и симптомах [16]. Шкала GOLD представляет собой визуальную аналоговую шкалу, на которой пациент отвечает на вопрос: «Всегда ли Вы чувствуете начало гипогликемии?» — варианты ответов варьируют от «1 — всегда чувствую» до «7 — никогда не чувствую» [15]. 4 и более баллов по опроснику Clarke и шкале GOLD расценивались как нарушение распознавания гипогликемии.

## Статистический анализ

Анализ данных проводился в программах SPSS Version 26.0 (IBM, США), Microsoft Excel 2016 (Microsoft, США), StatTech v. 4.6.3 (разработчик — ООО «Статтех», Россия). Количественные показатели оценивались на предмет соответствия нормальному распределению с помощью критерия Шапиро-Уилка (при числе исследуемых менее 50) или критерия Колмогорова-Смирнова (при числе исследуемых более 50). Количественные показатели, имеющие нормальное распределение, описывались с помощью средних арифметических величин (M) и стандартных отклонений (SD), границ 95% доверительного интервала (95% ДИ). В случае отсутствия нормального распределения количественные данные описывались с помощью медианы (Me) и нижнего и верхнего квартилей (Q1–Q3). Сравнение двух групп по количественному показателю, распределение которого отличалось от нормального, выполнялось с помощью U-критерия Манна-Уитни. При сравнении количественных показателей, распределение которых отличалось от нормального, в двух связанных группах использовался критерий Уилкоксона. При сравнении трех и более зависимых совокупностей, распределение которых отличалось от нормального, использовался непараметрический критерий Фридмана с апостериорными сравнениями с помощью критерия Коновера-Имана с поправкой Холма. Различия считались статистически значимыми при p<0,05.

## Этическая экспертиза

Проведение исследования было одобрено комитетом по этике при ФГБОУ ВО Тюменского ГМУ Минздрава России от 28 ноября 2022 г. (выписка из протокола №110).

## РЕЗУЛЬТАТЫ

В основную группу было включено 107 человек с СД1, однако в процессе исследования 3 из них были исключены в связи с переходом на помповую инсулинотерапию, 12 — в связи с отзывом добровольного информированного согласия, 4 перестали выходить на связь. Таким образом, во всех процедурах, предусмотренных протоколом, приняли участие 88 человек, соответствующие критериям включения и не имеющие критериев исключения. Характеристика пациентов представлена в таблице 1, статистически значимых различий между параметрами основной и контрольной групп не выявлено (p>0,05).

**Table:** Таблица 1. Общие характеристики групп, вошедших в исследование

Параметры	Группа наблюдения	p
Основная группа(n=88)	Группа контроля(n=20)
Возраст (лет),М±SD (95% ДИ)	35,9±11,9(33,4–38,4)	38,5±13,9(32,0–44,9)	0,403
Пол:			
мужчины, n (%)	55 (62,5%)	10 (50,0%)	0,303
женщины, n (%)	33 (37,5%)	10 (50,0%)
Длительность СД (лет),Ме [ Q1; Q3]	13,0[ 7,8; 21,2]	15,0[ 11,0; 27,0]	0,164
Индекс массы тела (кг/м²),Me [ Q1; Q3]	23,4[ 21,9; 26,7]	26,0[ 24,6; 26,7]	0,140
Исходный HbA1c (%),Ме [ Q1; Q3]	9,0[ 8,4; 9,9]	9,6[ 8,9; 10,2]	0,101

Таким образом, в исследование вошли относительно молодые пациенты, преимущественно женщины, с длительностью СД1 более 10 лет; часть исследуемых имела избыточный вес. Исходный HbA1c был выше 9,0%.

За 6 месяцев исследования для пациентов основной группы было проведено 3 очных визита и в среднем 4,4±2,3 телеконсультации в квартал (в формате телефонных звонков или чатов), а для пациентов контрольной группы — 3 очных визита.

## Гликемический контроль

Динамика HbA1c в зависимости от группы наблюдения представлена на рисунке 2. Доля пациентов, достигших HbA1c менее 7,5% через 6 месяцев в основной группе, составила 29,5% (n=26), а в группе контроля — 5,0% (n=1) (p=0,022), HbA1c менее 7,0% достигли лишь 12,5% (n=11) (p=0,211) из группы вмешательства.

**Figure:**
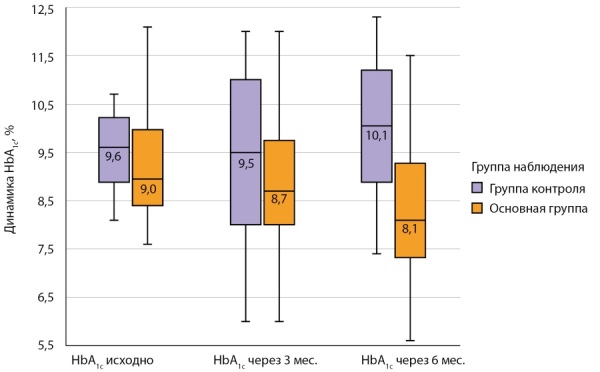
Рисунок 2. Анализ динамики HbA1c в зависимости от группы наблюдения, Me [ Q1; Q3], %.

В связи с несоблюдением частью участников основной группы рекомендуемой частоты СКГ в ходе исследования было принято решение разделить пациентов на группы по количеству измерений в сутки: 1) 1–2 раза в сутки (26,1%, n=23); 2) 2–4 раза в сутки (28,4%, n=25); 3) более 4 раз в сутки (45,5%, n=40). Важно отметить статистически значимые различия (p=0,042) в частоте СКГ по половому признаку: среди лиц женского пола СКГ более 4 раз в сутки проводили 54,5% (n=30), среди мужского пола — лишь 30,3% (n=10) пациентов. Было выявлено значительное статистически значимое снижение HbA1c на 1,4% (р<0,001) к концу исследования в третьей группе в сравнении с первой и второй группами. Результаты представлены на рисунке 3.

**Figure:**
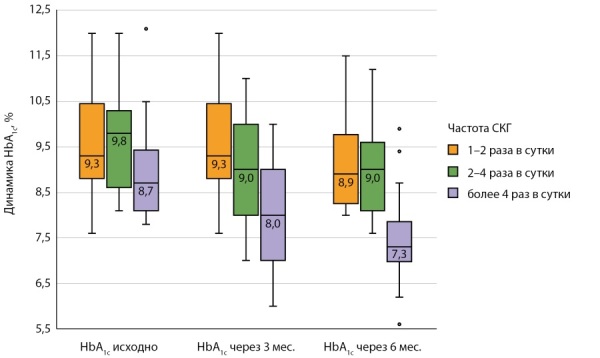
Рисунок 3. Динамика уровня HbA1c основной группы в зависимости от частоты СКГ, Me [ Q1; Q3], %.

В связи с тем, что согласно исследованиям наблюдается соответствие результатов времени нахождения в целевых диапазонах и вариабельности гликемии, полученных на основании НМГ и СКГ более 4 раз в сутки [17], в данной группе пациентов (n=40) нами был проведен анализ динамики расчетных показателей времени нахождения в целевых диапазонах и вариабельности гликемии за 6 месяцев (табл. 2).

**Table:** Таблица 2. Сравнительная характеристика динамики показателей контроля углеводного обмена по данным дистанционного мониторинга СКГ *Значимость различий между группами (различия статистически значимы при p<0,05).

Показатели	Этапы наблюдения	p
Исходно(n=40)	Через 6 месяцев(n=40)
dTIR,M±SD (95% ДИ)	52,70±18,38(46,82–58,58)	69,88±12,99(65,73–74,03)	<0,001*
dTAR,Me [ Q1; Q3]	37,0[ 23,8; 45,0]	9,5[ 6,4; 15,0]	<0,001*
dTBR,Me [ Q1; Q3]	9,1[ 5,0; 14,2]	6,7[ 2,8; 12,2]	0,033
CV,M±SD (95% ДИ)	43,7±8,4(41,1–46,4)	36,3±7,9(33,7–38,8)	<0,001*

## Качество жизни

Результаты анализа анкет по качеству жизни SF-36 представлены в таблице 3. Статистически значимых различий исходных параметров между группами не выявлено (p>0,05). Через 6 месяцев в основной группе отмечалось статистически значимое улучшение физического и психологического компонентов качества жизни, в частности за счет параметров общего состояния здоровья (GH), жизненной активности (VT), социального функционирования (SF), ролевого эмоционального функционирования (RE) и психического здоровья (MH). В группе контроля не удалось выявить статистически значимых изменений (p>0,05).

**Table:** Таблица 3. Сравнительный анализ динамики параметров качества жизни по опроснику SF-36, Me [ Q1; Q3] *Значимость различий между группами (различия статистически значимы при p<0,05).

Параметры качества жизни	Этапы наблюдения	p
Исходно	Через 6 месяцев
Основная группа	Группа контроля	Основная группа	Группа контроля
Физический компонент здоровья
Физическое функционирование, PF	95,0[ 75,0; 100,0]	87,5[ 67,5; 95,0]	95,0[ 80,0; 100,0]	78,0[ 62,8; 86,2]	P1=0,179P2=0,364
p	0,226	0,007*	
Ролевое физическое функционирование, RP	100,0[ 50,0; 100,0]	87,5[ 50,00; 100,00]	100,0 [ 50,0; 100,0]	75,00[ 55,75; 100,0]	P1=0,196P2=0,893
p	0,745	0,309	
Интенсивность боли, BP	80,0[ 52,0; 100,0]	87,0[ 74,0; 100,0]	74,0[ 62,0; 100,0]	74,0[ 68,0; 100,0]	P1=0,540P2=0,456
p	0,450	0,725	
Общее состояние здоровья, GH	57,0[ 44,2; 72,0]	57,0[ 47,5; 61,2]	61,0[ 45,0; 77,0]	55,5[ 45,0; 77,0]	P1=0,013*P2=0,211
p	0,454	0,376	
Психологический компонент здоровья
Жизненная активность, VT	65,0[ 50,0; 75,0]	50,0[ 38,8; 70,0]	70,0[ 60,0; 85,0]	52,5[ 43,0; 70,0]	P1<0,001*P2=0,150
p	0,112	0,001*	
Социальное функционирование, SF	87,50[ 62,50; 100,0]	81,25[ 46,88; 87,50]	100,00[ 75,00; 100,0]	67,50[ 43,75; 86,25]	P1=0,004*P2=0,078
p	0,087	< 0,001*	
Ролевое эмоциональное функционирование, RE	96,0[ 33,3; 100,0]	84,5[ 66,0; 100,0]	73,0[ 54,2; 100,0]	100,0[ 66,9; 100,0]	P1=0,013*P2=0,706
p	0,796	0,093	
Психическое здоровье, MH	68,0[ 56,0; 76,0]	76,0[ 60,0; 84,0]	78,0[ 64,8; 84,0]	68,0[ 52,0; 77,0]	P1<0,001*P2=0,121
p	0,163	0,045*	
Физический компонент здоровья, PH	51,25[ 46,12; 55,52]	50,95[ 42,50; 53,85]	49,25[ 45,9; 55,70]	54,50[ 47,75; 55,98]	P1=0,931P2=0,177
p	0,646	0,309	
Психологический компонент здоровья, MnH	48,80[ 39,83; 53,40]	46,74[ 44,80; 53,32]	53,80[ 49,22; 57,00]	55,00[ 49,22; 55,92]	P1<0,001*P2=0,064
p	0,956	0,940	

Анализ нарушения распознавания гипогликемии по результатам опросника Clarke и шкалы GOLD представлен в таблице 4. В основной группе выявлено статистически значимое уменьшение количества пациентов с нарушением распознавания гипогликемии, в группе контроля достоверных изменений не выявлено.

**Table:** Таблица 4. Сравнительный анализ наличия нарушения распознавания гипогликемии в динамике *Значимость различий между группами (различия статистически значимы при p<0,05).

Группа наблюдения	Этапы наблюдения	p
Исходно	Через 6 месяцев
Шкала GOLD
Основная группа, n (%)	18 (20,5)	11 (12,5)	0,008*
Группа контроля, n (%)	5 (25)	5 (25)	-
p	0,763	0,171	
Опросник Clarke
Основная группа, n (%)	11 (12,5)	7 (8,0)	0,046*
Группа контроля, n (%)	3 (15,0)	2 (10,0)	0,317
p	0,721	0,671	

## ОБСУЖДЕНИЕ

## Репрезентативность выборок

Репрезентативность выборки позволяет сделать выводы по настоящему клиническому исследованию и может экстраполироваться на целевую популяцию — пациентов с СД1, так как набор участников исследования проводился в нескольких лечебно-профилактических учреждениях по территориальному принципу.

## Сопоставление с другими публикациями

Многочисленные когортные исследования показывают прямую связь между частотой СКГ и гликемическим контролем [18][19]. Систематический обзор и метаанализ эффективности мобильных приложений, в том числе дистанционного мониторинга СКГ, для пациентов с СД выявляет благоприятные результаты в контроле HbA1c, а также положительные и статистически значимые изменения в качестве жизни и удовлетворенности лечением в группе вмешательства [20][21].

В нашем исследовании продемонстрировано статистически значимое снижение HbA1c в группе дистанционного мониторинга СКГ. Полученные результаты сопоставимы с выводами Charpentier G. и соавт. В 6-месячном открытом многоцентровом исследовании рандомизировали взрослых пациентов с СД1 (n=180) с уровнем HbA1c более или равным 8,0% (9,07±1,07%) в группы обычного ежеквартального наблюдения (G1), домашнего использования смартфона с рекомендациями доз инсулина при ежеквартальных визитах (G2) и использования смартфона с короткими телеконсультациями каждые 2 недели, но без визитов до конечной точки (G3). HbA1c через 6 месяцев в группе G3 (8,41±1,04%) был ниже, чем в группе G1 (9,10±1,16%; p=0,002). В группе G2 были получены промежуточные результаты (8,63±1,07%). Дистанционный СКГ способствовал улучшению HbA1c на 0,91% [ 0,60; 1,21] по сравнению с контрольной группой и снижение на 0,67% [ 0,35; 0,99] при использовании мобильного дневника СКГ без телеконсультации. Однако, как и по результатам нашего исследования, лишь малая доля пациентов G3 (17%) достигла HbA1c менее 7,5% через 6 месяцев [20].

Основой тактики ведения больных с нарушением распознавания гипогликемии является профилактика новых эпизодов гипогликемии [22]. В ходе нашего исследования было выявлено благоприятие воздействие дистанционного мониторинга данных СКГ на распознавание гипогликемии. Вероятнее всего, это связано с большей частотой СКГ, улучшением показателей контроля углеводного обмена, а также анализом предыдущих эпизодов гипогликемии, своевременной коррекцией доз инсулина, психологической поддержкой врача-эндокринолога. Положительное влияние дистанционного СКГ на качество жизни пациентов с СД1 по результатам опросника SF-36 в группе вмешательства определенно ассоциировано с достижением целей гликемического контроля, повышением вовлеченности и мотивации пациента, что сопоставимо с исследованием Rossi M.C. и соавт. [23].

По всей видимости, недостижение индивидуальных целевых показателей углеводного обмена через 6 месяцев связано с тем, что исследование проводилось на пациентах с потерей гликемического контроля и высоким исходным значением HbA1с. Обращает на себя внимание значительная доля участников основной группы, выбывших по причине отказа от дальнейшего участия или потери связи с пациентом -15% (n=16), несоблюдение рекомендованной частоты СКГ, что, безусловно, связано с психолого-поведенческими характеристиками пациентов с СД1 и требует дальнейшего изучения.

## Клиническая значимость результатов

Клиническая значимость результатов исследования дает возможность широкого использования дистанционного мониторинга СКГ в амбулаторной практике.

## Ограничения исследования

Ограничение исследования — невключение пациентов с СД1 на помповой инсулинотерапии.

## Направления дальнейших исследований

Изучение влияния дистанционного мониторинга СКГ на углеводный обмен и качество жизни пациентов с СД2 является актуальным для будущих исследований. Представляется перспективным составление портрета пациента, для которого дистанционный мониторинг СКГ будет наиболее эффективным.

## ЗАКЛЮЧЕНИЕ

Положительное влияние дистанционного мониторинга СКГ на углеводный обмен и качество жизни пациентов с СД1, доступность данной технологии свидетельствует о потенциальной перспективности широкого ее применения среди пациентов с СД. Несоблюдение рекомендованной частоты СКГ пациентами с СД1, значительный процент отказа основной группы от участия в исследовании, низкий процент достижения целевого HbA1c, исходно низкий показатель психологического компонента качества жизни по результатам опросника SF-36 указывают на сложность ведения данной категории пациентов, необходимость разработки новой стратегии по достижению целевых показателей углеводного обмена, в том числе, вероятно, с использованием психотерапевтических подходов.

## ДОПОЛНИТЕЛЬНАЯ ИНФОРМАЦИЯ

Источники финансирования. Работа выполнена по инициативе авторов без привлечения финансирования.

Конфликт интересов. Авторы декларируют отсутствие явных и потенциальных конфликтов интересов, связанных с содержанием настоящей статьи.

Благодарности. Авторы выражают благодарность медицинским организациям, участвующим в реализации пилотного проекта в г. Тюмени, врачам-эндокринологам, принимающим непосредственное участие в дистанционном мониторинге СКГ: Бородулиной Оксане Геннадьевне, Пруцковой Наргисе Абдулакимовне, Матюшкиной Екатерине Александровне, Куртековой Ирине Владимировне, Киршановой Людмиле Александровне, Валенюк Алене Николаевне, Сало Светлане Сергеевне, Патрушевой Маргарите Ильиничне, Пимшиной Ларисе Анатольевне, Когут Юлии Павловне, Мойсиевой Ольге Михайловне, Горбуновой Анастасии Александровне. Авторы признательны за техническую поддержку проекта специалисту ГАУЗ ТО «Городская поликлиника №8» Петриченко Дмитрию Александровичу, а также сотрудникам компании «ЭЛТА».
